# Pure electron-electron dephasing in percolative aluminum ultrathin film grown by molecular beam epitaxy

**DOI:** 10.1186/s11671-015-0782-x

**Published:** 2015-02-18

**Authors:** Shih-Wei Lin, Yue-Han Wu, Li Chang, Chi-Te Liang, Sheng-Di Lin

**Affiliations:** Department of Electronics Engineering, National Chiao Tung University, 1001 University Road, Hsinchu, 30010 Taiwan; Department of Materials Science and Engineering, National Chiao Tung University, Hsinchu, 30010 Taiwan; Department of Physics, National Taiwan University, Taipei, 10617 Taiwan; Geballe Laboratory for Advanced Materials (GLAM), Stanford University, Stanford, CA 94305 USA

**Keywords:** Weak anti-localization, Ultrathin metal films, Pure electron-electron dephasing

## Abstract

We have successfully grown ultrathin continuous aluminum film by molecular beam epitaxy. This percolative aluminum film is single crystalline and strain free as characterized by transmission electron microscopy and atomic force microscopy. The weak anti-localization effect is observed in the temperature range of 1.4 to 10 K with this sample, and it reveals that, for the first time, the dephasing is purely caused by electron-electron inelastic scattering in aluminum.

## Background

Weak localization (WL) is the quantum correction to the conductance which occurs in weakly disordered systems due to coherent backscattering of electrons (or holes). As a results of spin-orbit coupling, weak anti-localization (WAL) may be observed in a weakly disordered electron (or hole) system [1]. The theoretic derivation and experimental proof of WAL were extensively developed since 1980s, and the investigation on various materials in all dimensions has been a central topic in condensed matter physics for decades. In particular, a wide variety of experimental results of WAL were obtained in two-dimensional (2D) systems. 2D system is suitable for experimental study of WAL because of its stronger WAL contribution than three-dimensional (3D) ones and its easier sample fabrication than one-dimensional (1D) ones. Recently, due to its sensitivity to the electron dephasing and spin dephasing, WAL has been widely applied to studying the spin-orbit interaction in new materials, such as graphene, topological insulator, magnetic-doped semiconductor, and narrow-gap semiconductor, to evaluate the potential for spintronics devices [2-7].

In addition, the interplay between superconducting effect and WAL also attracts much attention and has been investigated extensively. Ebisawa et al. derived the relationship between the superconducting pair-breaking parameter *δ* and the inelastic scattering rate *τ*_i_^−1^ by *δ* = (*πℏ*/8*k*_B_*T*)*τ*_i_^−1^ [8]. Their results have been used to study experiments of WAL in aluminum thin films [9-11]. However, it is a chanting challenge to grow an ideal 2D superconducting metallic sample. As the thickness of evaporated metal goes thinner, the discontinuity of the metal film un-avoidably appears due to large lattice mismatch between the template and metallic material as well as the surface non-uniformity of the bottom template. Previously, the reported metallic films were in the thickness of ten to a few tens of nanometers [9-14]. Although plenty of the theoretic works of WAL in two-dimensional systems have been published in the past few decades, the experimental proof toward WAL in an ideal two-dimensional metallic system is still lacking. In this work, we have used molecular beam epitaxy (MBE) system as the deposition technique to prepare ultrathin Al films. By using gallium-rich GaAs as the epi-template, we are able to successfully deposit ultrathin percolated Al film for studying WAL toward the 2D limit. Interestingly, we have observed a pure electron-electron dephasing in this sample over the whole temperature range that WAL effect exists. Note that all of our characterizations including structural and electrical assessments were fulfilled *ex situ*, and the continuity of the Al film remains even after the post-processing for the Hall device fabrication. Even though our sample is not thin enough to reach the ultimate two-dimensional limit such as monolayer graphene, our results still provide an experimental proof that Nyquist scattering becomes the dominant inelastic scattering mechanism at all temperatures when the system approaches an ideal two-dimensional one.

### Theory

Magneto-resistance measurements are commonly used to study WAL. The theoretical calculation of 2D WAL in a perpendicular magnetic field was derived by Hikami, Larkin, and Nagaoka [1]. The difference of conductance induced by applied magnetic field can be expressed as:

1 $$ \Delta {g}_{\mathrm{WAL}}=g(B)-g(0)=\frac{e^2}{\pi h}\left[\frac{3}{2}Y\left(\frac{B_2}{B}\right)-\frac{1}{2}Y\left(\frac{B_{\mathrm{i}}}{B}\right)\right]. $$

where *B* is the applied magnetic field. *Y*(*x*) represents *Ψ*(1/2 *+ x*) *−* ln(*x*), *B*_2_ *= B*_i_ *+* 3/4*B*_so_, *Ψ*(*x*) is the digamma function. Here, *B*_i_ and *B*_so_ represent the strength of electron dephasing and spin-orbit interaction, respectively. For a superconducting material near its critical temperature *T*_c_, superconducting fluctuations must be considered. Maki has calculated the effect of superconducting fluctuations in a 2D system (Δ*g*_MT_) [15]. Later, Thompson has modified the model by introducing the superconductor pair-breaking parameter *δ* to avoid the unphysical divergence at temperature near *T*_c_ [16]. The Maki-Thompson correction term was modified by Abrahams et al. that can be applied to higher field [17]:

2 $$ \Delta {g}_{\mathrm{MT}}=-\beta \frac{e^2}{\pi h}\left[Y\left(\frac{B_{\mathrm{i}}}{B}\right)-Y\left(\frac{B_{\mathrm{T}}}{B}\right)\right], $$

where *β* represents the interaction strength between the electron pair [18]. Accordingly, *β ~* 1/[ln(*T*/*T*_c_) *− δ*] at temperature close to *T*_c_ [17]. *B*_T_ *=* 2*k*_B_*T*ln(*T*/*T*_c_)*/πDe* and *D* is the diffusion coefficient, *D* = 1/3*v*_F_*l*_0_. Here, *v*_F_ is the Fermi velocity and *l*_0_ is the mean free path.

Bergmann experimentally studied WAL in various metallic thin films [19] and confirmed that the Maki-Thompson correction gives the most contribution of superconducting fluctuations and cannot be neglected even for temperatures far above *T*_c_. Therefore, in our following analysis, both WAL effect and Maki-Thompson term will be taken into account.

## Methods

### Sample fabrication

The epitaxial aluminum thin film was fabricated on a semi-insulating gallium arsenide (SI-GaAs) substrate in our Varian Gen II MBE system equipped with an arsenic cracking cell. First, a GaAs wafer was heated to 620°C for 20 min for de-oxidation under As flux, and then a 300-nm-thick undoped GaAs buffer layer was deposited at 590°C as the epitaxial template. The sample surface was turned into Ga-rich at 620°C in the absence of As flux. We kept the sample in high vacuum (approximately 3 × 10^−10^ Torr) until the background As vapor was pumped out. The Al film with an intentional thickness of 3 nm was then deposited at room temperature with a growth rate of 0.366 μm/h.

The 50-μm-wide Hall devices were processed using conventional lithography technique. Al was etched by 2% tetramethylammonium hydroxide (TMAH) for 20 s to transfer the Hall bar pattern from photoresist to our Al film. Ti/Au (30/300 nm) was deposited using e-gun evaporation as the contact electrode. The finished devices were wire-bonded on a ceramic carrier and loaded into ^4^He cryogenic system equipped with a superconducting magnet. A DC four-terminal electrical measurement has been performed in this work for studying WAL. We have used a Keithley 2602 multi-meter (Keithley Instruments Inc., Cleveland, OH, USA) as a constant current source as well as a voltage meter. Electrical current was set at 3 μA for clear signals and was low enough in order to avoid possible current heating effect. Voltage noise level was at about 5 μV.

## Results and discussion

**Figure 1:**
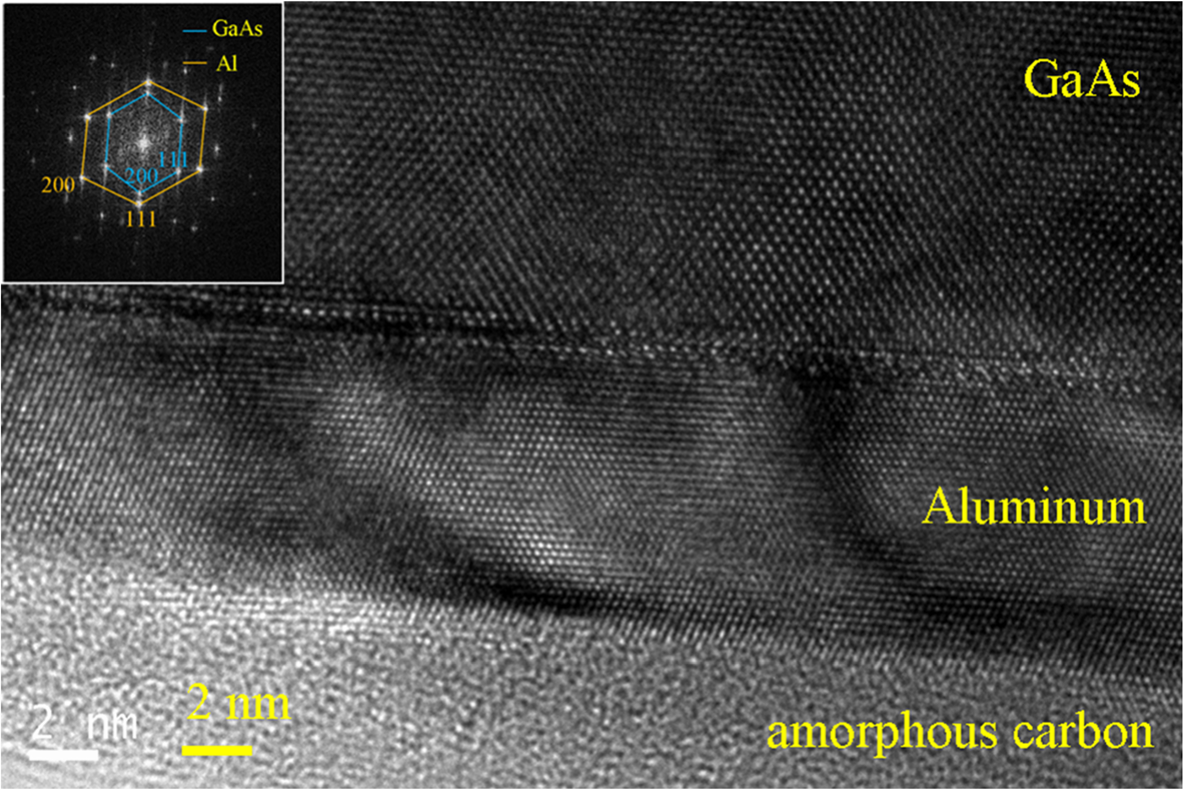
**Cross-sectional TEM image of the Al sample.** GaAs/Al interface in the middle and the native oxide of Al at the bottom are clearly spotted. The upper left inset is the diffraction pattern showing the GaAs (Al) diffracted spots in the inner (outer) hexagon.

**Figure 2:**
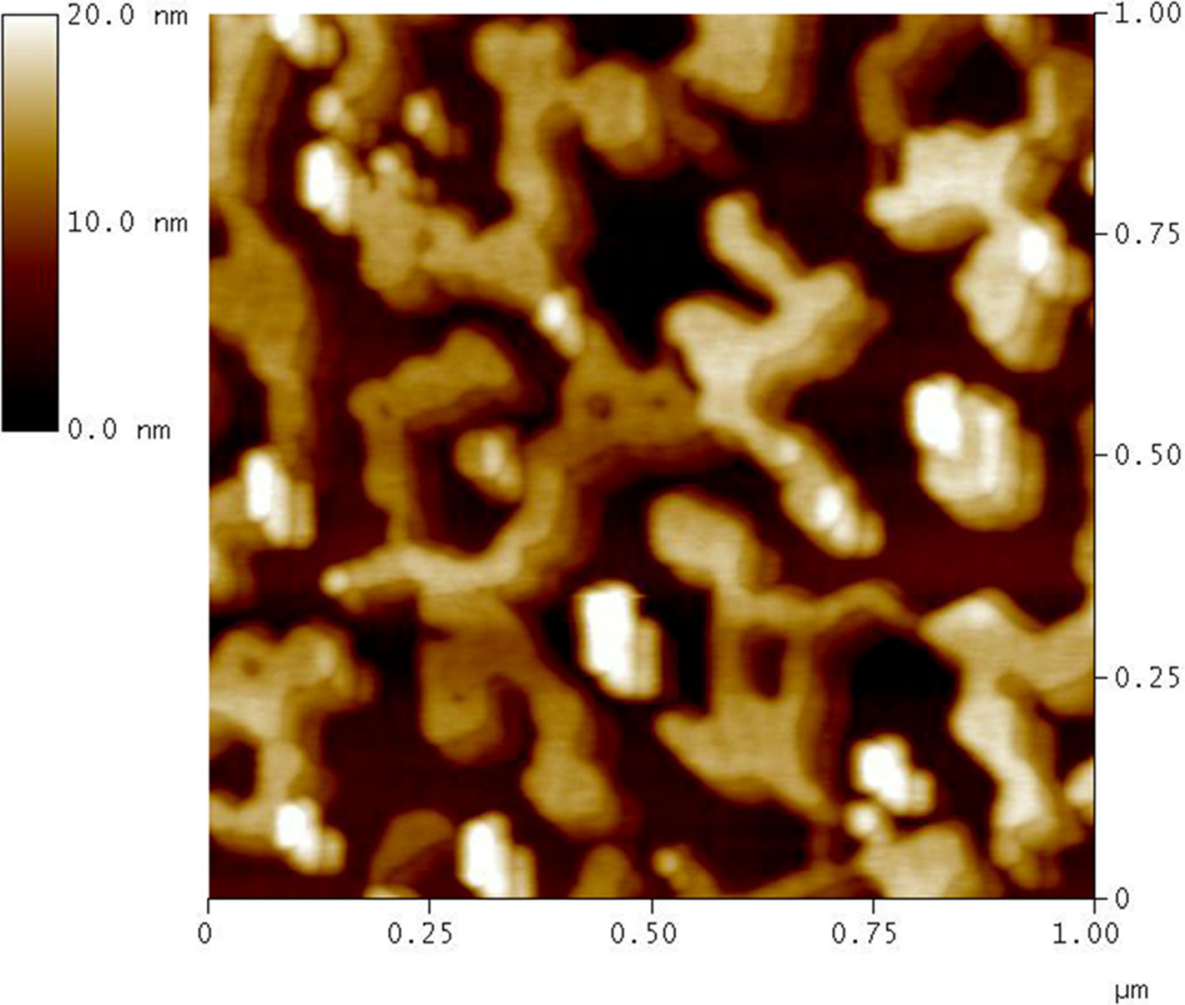
**The 1 × 1 μm** ^**2**^ **AFM image of the Al sample showing the sample roughness is about 4.9 nm.**

We have used cross-sectional high-resolution transmission electron microscopy (TEM) and atomic force microscopy (AFM) for the investigation of film crystal quality and surface morphology. Figure 1 shows the TEM image of our epitaxial Al film taken with electron beam along the $<0\overline{1}1{>}_{\mathrm{GaAs}}$ zone axis. A clear interface between the GaAs template and deposited Al can be seen in the middle of the picture. At the bottom of Figure 1, we can see an amorphous layer which may be amorphous carbon from TEM specimen preparation. The thickness of Al layer is about 8 nm which is thicker than the deposited amount because our Al film is percolative as we shall see later in the AFM images. A detailed examination of the TEM image at the interface can show the existence of misfit dislocations, implying that most of the strain in the Al film caused by lattice mismatch between Al and GaAs is released. The inset of Figure 1 is the fast Fourier transform diffraction pattern taken around the interface between GaAs and Al. Clear diffraction spots can be seen. The inner hexagon is the diffraction spots of the bottom GaAs template, and the outer one is for epi-aluminum film, indicating that the epitaxial relationship of Al with GaAs is (100)_GaAs_ // (111)_Al_, <011 > _GaAs_ // $<\overline{2}11{>}_{\mathrm{Al}}$, and $<0\overline{1}1{>}_{\mathrm{GaAs}}$ // $<0\overline{1}1{>}_{\mathrm{Al}}$; the last axis is also the observation plane of the TEM image. The axis arrangement is different from the previous works [20-22] probably due to the different surface conditions used. Clear spots indicating good crystal quality and no deformation of the diffraction spots are observed even the strain accumulation due to lattice mismatch could occur in our sample. Figure 2 shows a 1 × 1 μm^2^ AFM image of our Al film. Obviously, a percolating but continuous morphology has been seen. This kind of morphology was generally observed when metal films are deposited onto a semiconductor or dielectric template because of poor affinity between them [23]. Although the bi-polarized atomic bonding of the bottom GaAs template or inhomogeneous surface atomic deformation could also play a role here, we have used a smooth Ga-rich surface as the epitaxial template to minimize these two issues. The roughness of the film is about 4.9 nm which is thicker than the deposited thickness and is possibly caused by the Al oxidation after the exposure to the air.

**Figure 3:**
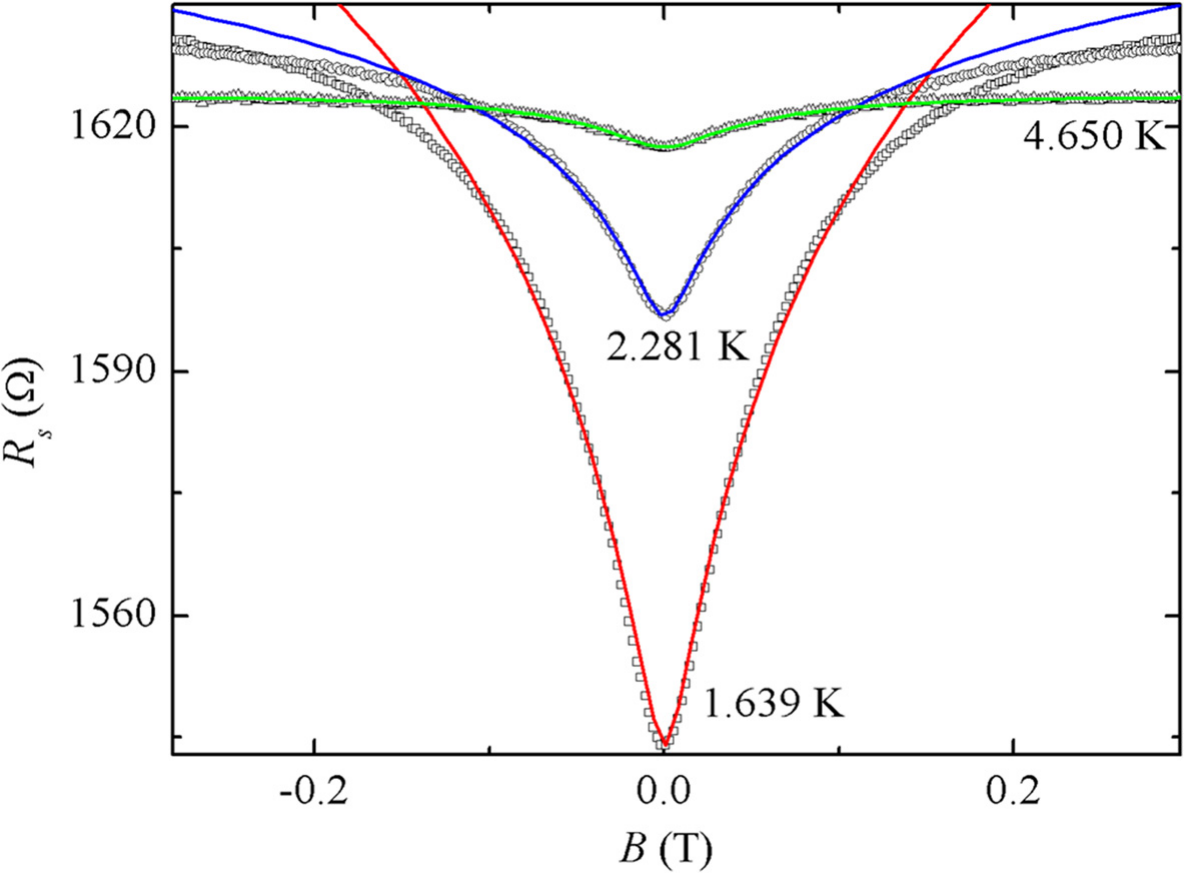
**Measured and calculated sheet resistance.** Measured (symbols) and calculated (solid lines) sheet resistance as a function of magnetic field of the Al film at 1.639, 2.248, and 4.650 K.

We have measured the sheet resistance *R*_s_ of our sample in the temperature range of 1.4 to 10 K. At temperatures higher than 10 K, the signals became noisy and WAL is barely observable. We noted that the Hall resistance *R*_H_ is small compared to *R*_s_ and remained unchanged even increasing the magnetic field to 1 T. Thus, we neglected the effect of *R*_H_ in all the calculation and theoretical fitting. In Figure 3, the measured *R*_s_ is plotted against the applied magnetic field at various temperatures, together with the fit to the theoretical model. Around zero magnetic field, clear WAL in our Al film occurs as *R*_s_ increases with increasing magnetic fields. In addition, *R*_s_ decreases dramatically when the temperature goes below 4.5 K, indicating that the superconducting fluctuation plays an important role here. Therefore, when fitting our data to the theoretical model, we have considered the WAL theory stated in Equation 1 with the first Maki-Thompson term in Equation 2. The later term of Equation 2 vanishes because *B*_T_ is always much larger than *B*_i_ and *B*_so_. The contribution of this term is much less. So, the used fitting formula is given by:

3 $$ \Delta g=\frac{e^2}{\pi h}\left[\frac{3}{2}Y\left(\frac{B_2}{B}\right)-\frac{1}{2}Y\left(\frac{B_i}{B}\right)-\beta Y\left(\frac{B_i}{B}\right)\right]. $$

In our fitting procedure, *B*_i_ and *β* were temperature-dependent fitting parameters. *B*_so_ was chosen as a temperature-invariant constant because it is commonly accepted that the electron configuration in the half-filled conduction bands is insensitive to the temperature, so the angular momentum and the spin-orbit interaction remains unchanged at all temperature in metals [10-13]. In Figure 3, the solid lines represent theoretic fits of our experimental data at three temperatures as an example. It is clear that the theory can only be applied to small magnetic field. At higher temperatures, the fits can be extended to higher magnetic fields. This is because the Maki-Thompson term is only valid at *B* ≪ *k*_B_(*T − T*_c_)/4*eD* [15-17].

**Figure 4:**
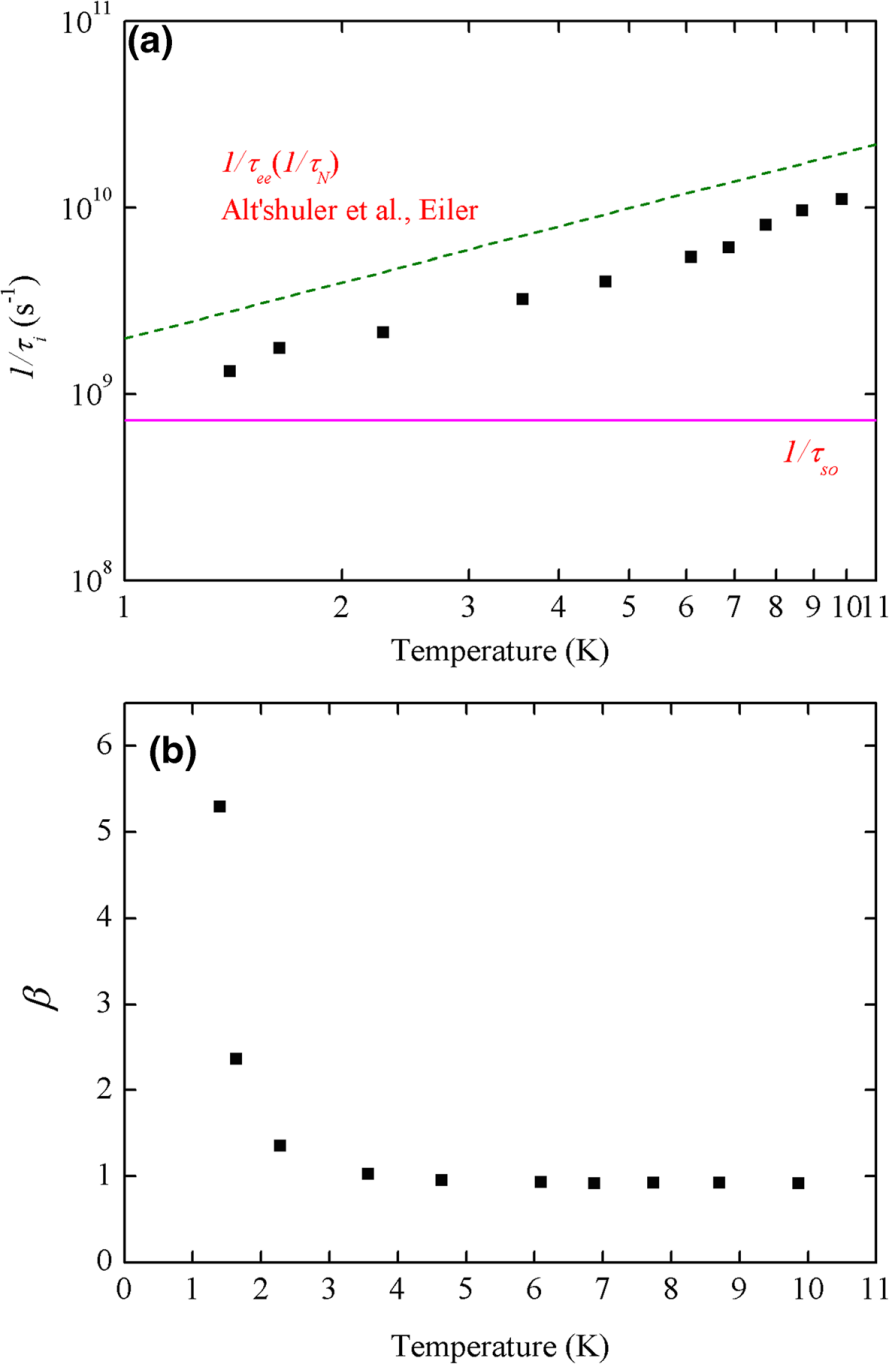
**Temperature-dependent dephasing rate and interaction strength. (a)** Temperature-dependent dephasing rate (1/*τ*_i_) (symbol) of the Al sample. The horizontal solid line represents the level of spin-orbit interaction rate (1/*τ*_so_) and the dashed line represents the theoretic Nyquist scattering rate (1/*τ*_N_). **(b)** Temperature dependence of the interaction strength *β*.

With the fitted *B*_i_ and *B*_so_, the inelastic scattering time *τ*_i_ and spin-orbit scattering time *τ*_so_ can be derived by *τ*_i_ = *h*/(8*πeDB*_i_) and *τ*_so_ = *h*/(8*πeDB*_so_), as well as the phase coherent length *l*_i_ and spin-orbit interaction length *l*_so_ by *l*_i_ = (*h*/8*πeB*_i_)^1/2^ and *l*_so_ = (*h*/8*πeB*_so_)^1/2^. We used Al bulk concentration *n*_Al_ = 1.81 × 10^29^ (m^−3^) to estimate the Fermi velocity (*v*_F_ = 2.03 × 10^6^ m/s) and Fermi wavelength (*λ*_F_ *=* 0.36 nm) for our sample. Figure 4a shows the extracted parameter 1/*τ*_i_ as a function of temperature. The solid horizontal line indicates the level of spin orbit interaction rate 1*/τ*_so_, and the dashed line represents the theoretic calculation of electron-electron scattering (Nyquist scattering) rate based on the work of Altshuler et al. [24]:

4 $$ {\tau_{\mathrm{N}}}^{-1}=\frac{e^2{R}_{\mathrm{s}}}{2\pi {\hslash}^2}{k}_{\mathrm{B}}T \ln \left(\frac{\pi \hslash }{e^2{R}_{\mathrm{s}}}\right). $$

In our 3-nm-thick aluminum sample, weak anti-localization changes into weak localization when the temperature goes above 10 K. In the temperature range for existing WAL (*T* < 10 K), we found that 1/*τ*_i_ is proportional to *T*, which agrees with the theoretic prediction of Nyquist scattering (dashed line) in the same order.

We would like to emphasize that our ultrathin Al film has a very high carrier concentration (*n* ~ 10^29^ m^−3^), so it is actually in mixed dimensions. That is, considering the density of states and diffusive motion, it is treated as a 3D system because the film thickness *d* is much larger than the Fermi wavelength *λ*_F_ (*d* ≫ *λ*_F_ *~* 10^−1^ nm) and its mean free path of electrons *l*_0_ (*d* ≫ *l*_0_ *~* 10^−1^ nm in our sample). However, in terms of electron dephasing, the film thickness is much less than the dephasing length *l*_i_ (*d* ≪ *l*_i_ ~ few tens of nanometers) so the system is two-dimensional. Previous experimental studies on clean and dirty Al thin films demonstrated that, only for the temperature near *T*_c_, Nyquist scattering was the major dephasing mechanism. When the temperatures were higher than about 4 K, electron-phonon scattering dominated the dephasing process [9-14]. In contrast, our results show a pure electron-electron dephasing all the way up 10 K. For a type I superconducting material, there is only one experimental result on clean titanium film exhibiting a full *T*^−1^ dependence of dephasing rate [25]. However, our sample is in the dirty limit as the sheet resistivity of our sample is over 1.6 kΩ, at least one order higher than those all of their samples. On the other hand, for type II superconductors such as ZrRh and TaN, most experimental works did not observe the pure *T*^*−*1^ dependence of dephasing rate [26,27]. It is worth mentioning that the observation of Giannouri et al. [28] with their NbTa film is very similar to ours except that the sudden drop of dephasing rate at temperature approaching *T*_c_ has not been seen even at 1.4 K in our experiment.

Dephasing process which purely comes from electron-electron scattering observed here is unusual but is relatively easy to be obtained in semiconductor-based 2D systems, such as 2D electron or hole gases (2DEG and 2DHG), formed by modulation-doped heterostructures. The carriers (electrons or holes) are confined by the triangular potential well caused by remote ionized dopants and limited in the few monolayers next to the heterostructure interface. The carrier concentration of 2DEG or 2DHG is controlled in the range of 10^10^ ~ 10^12^ cm^−2^, so the Fermi wavelength *λ*_F_ easily exceeds the size of the confinement potential well and the system is quantized in this dimension to become two-dimensional. However, for metallic materials even down to a few nanometers, the carrier concentration is still as high as 10^15^ cm^−2^, which makes the *λ*_F_ is much smaller than its thickness. Recently, the experimental results of WAL in various semiconductor heterostructures were published, including AlGaN/GaN, GaAs/InGaAs, InP/InGaAs, and AlGaAs/GaAs [29-35]. Most of these works demonstrated a full *T*^−1^ dependence of the dephasing rate up to 10 K and considered as consistent with the theoretic calculation of Nyquist scattering because of their 2D nature. In our Al film, the observed pure 2D-like dephasing indicates that the superconducting metallic film can be an ideal 2D system in inelastic process.

We wish to address the issue of zero-temperature saturation rate (1*/τ*_i_^0^) that is the dephasing rate in the zero temperature limit. The origin of the saturation rate is still under debate for the time being [36]. Some of the reported experimental works on superconducting metallic materials revealed a divergence dephasing rate when the temperature approached *T*_c_ [9,10,13,26,27]. In contrast, such situations have not been observed in our sample that exhibits a full Nyquist scattering rate from liquid helium temperatures to 10 K, thus could be helpful for developing the related theory.

In Figure 4b, the electron-electron interaction strength parameter *β* in the Maki-Thompson correction term is plotted as a function of temperature. It is clear that *β* has a trend to diverge at temperature approaching *T*_c_, as expected with Larkin’s theory [18]. The value of our sample decreases dramatically with the increasing temperature because the superconducting effect becomes less significant. It is noted that *β* does not vanish but converge to about 0.9 instead. A similar result was observed in ZrRh films [26]. Further investigations on this non-zero *β* are certainly needed.

We note that the sheet resistivity of our sample is about 1.6 kΩ. The mean free path *l*_0_ actually is 0.082 nm estimated by the Drude model, which is slight shorter than *λ*_F_. This appears to contradict to the basic assumption of the WAL theory that WAL occurs in a weakly disordered system (*l*_0_ ≫ *λ*_F_). In a strongly disordered system, WAL should not be observed. We believe that, due to the percolative morphology of our sample, the actual electron path is much longer than the Hall bar size; therefore, the mean free path *l*_0_ has been underestimated.

Similar to the WAL effect, the universal conductance fluctuations (UCFs) due to the electron (or hole) interference between two classical paths is closely related to the phase coherent length *l*_i_ [37-40]. We have also examined the conductance fluctuations in our measured data, but no clear UCFs are observed, which could be due to the large size of our device and the limit of the measurement system noise (approximately 5 μV).

## Conclusions

We have presented the structural and electrical characterization of the ultrathin percolating aluminum film grown by MBE. The TEM results indicate a superior crystal quality of the epitaxial aluminum film. WAL revealed by low-temperature magnetoresistance measurement showed its unusual dephasing mechanism. At all temperatures that WAL exists, a pure electron-electron scattering was observed, so the aluminum film behaves as an ideal two-dimensional system in this aspect. Based on this, we conclude that the MBE-grown aluminum films could achieve the two-dimensional limit of a superconducting metallic material.
